# Establishment of patient-derived cancer xenografts in immunodeficient NOG mice

**DOI:** 10.3892/ijo.2015.2997

**Published:** 2015-05-11

**Authors:** TSUYOSHI CHIJIWA, KENJI KAWAI, AKIRA NOGUCHI, HIDEMITSU SATO, AKIMUNE HAYASHI, HARUHIKO CHO, MANABU SHIOZAWA, TAKESHI KISHIDA, SOICHIRO MORINAGA, TOMOYUKI YOKOSE, MAKOTO KATAYAMA, NOBUO TAKENAKA, HIROSHI SUEMIZU, ROPPEI YAMADA, YOSHIYASU NAKAMURA, TAKASHI OHTSU, YASUO TAKANO, KOHZOH IMAI, YOHEI MIYAGI, MASATO NAKAMURA

**Affiliations:** 1 Central Institute for Experimental Animals, Tonomachi, Kawasaki-ku, Kawasaki, Kanagawa 210-0821, Japan; 2 Department of Pathology, Kanagawa Cancer Center, Nakao, Asahi-ku, Yokohama 241-8515, Japan; 3 Department of Neurosurgery, Kanagawa Cancer Center, Nakao, Asahi-ku, Yokohama 241-8515, Japan; 4 Department of Gastrointestinal Surgery, Kanagawa Cancer Center, Nakao, Asahi-ku, Yokohama 241-8515, Japan; 5 Department of Urology, Kanagawa Cancer Center, Nakao, Asahi-ku, Yokohama 241-8515, Japan; 6 Research Institute, Kanagawa Cancer Center, Nakao, Asahi-ku, Yokohama 241-8515, Japan; 7 Department of Neurosurgery, Kawasaki Municipal Hospital, Shinkawa-tori, Kawasaki 210-0013, Japan; 8 Department of Regenerative Medicine, Tokai University School of Medicine, Shimokasuya, Isehara, Kanagawa 259-1193, Japan

**Keywords:** patient-derived xenograft, NOG mice, tumor model, cancer xenopatients, anticancer therapies

## Abstract

Viable and stable human cancer cell lines and animal models combined with adequate clinical information are essential for future advances in cancer research and patient care. Conventional *in vitro* cancer cell lines are commonly available; however, they lack detailed information on the patient from which they originate, including disease phenotype and drug sensitivity. Patient-derived xenografts (PDX) with clinical information (so-called ‘cancer xenopatients’) are a promising advance that may accelerate the development of anticancer therapies. We established 61 PDX lines from 116 surgically removed tumor tissues inoculated subcutaneously into NOG mice (53% success rate). PDX lines were established from various types of epithelial tumors and also from sarcomas, including gastrointestinal stromal tumors and Ewing/PNET sarcomas. The metastatic tumors yielded PDX lines more effectively (65%) than the primary tumors (27%, P<0.001). In our PDX models, morphological characteristics, gene expression profiles, and genetic alteration patterns were all well preserved. In eight cases (7%), the transplantable xenografts for several generations were composed of large monotonous nonepithelial cells of human origin, revealed to be Epstein-Barr virus infection-associated lymphoproliferative lesions. Despite this, PDX linked with clinical information offer many advantages for preclinical studies investigating new anticancer drugs. The fast and efficient establishment of individual PDX may also contribute to future personalized anticancer therapies.

## Introduction

Animal models have been used in front-line preclinical studies for predicting efficacy and possible toxicities of anticancer drugs in cancer patients (1,2). Current tumor models used for drug evaluation generally consist of implantation into immunodeficient mice of xenografts generated from well-established human cancer cell lines that have already adapted to *in vitro* growth. These models have been used extensively for decades for rapid screening of anticancer drug efficacy (3,4).

In recent years, xenografts derived from engrafting fresh surgical specimens directly into immunodeficient mice have enabled the development of more relevant *in vivo* models for human tumors (5). Such patient-derived xenograft (PDX) models, established by direct transfer of tumor tissue, retain similar morphology, architecture, and molecular signatures as the original cancers and thus should be used for rapid screening of potential therapeutics (6,7). Whereas the conventional xenograft models using cell lines provide only a monoclonal mass of tumor cells, PDX models recapitulate not only interactions from the host microenvironments but also the cancerous heterogeneity including the cancer stem cells (5,6,8). Results from these investigations support the use of direct transfer xenografts as a reliable strategy to anticipate clinical findings, provide direction for optimizing personalized treatment in advanced cancers, and suggest novel treatment opportunities in patients with no other therapeutic options (9). The advantages of PDX models in preserving cancer stem cells and the clinical information of the donor patient (so-called ‘cancer xenopatient’) may allow for accelerated cancer research by simulating the situation in cancer patients more closely (6,7).

However, the establishment of direct xenografts is still technically difficult (1,10,11). Recently, a new immunodeficient animal model, NOD/Shi-scid/IL-2Rγ^null^ (NOG) mice, derived from the NOD/SCID mouse with a common gamma chain, has been introduced. In addition to lacking functional T and B lymphocytes, the NOG mouse has multifunctional defects in natural killer cell activity, macrophage function, complement activity, and dendritic cell function (12). NOG mice were reported to be the most appropriate immunodeficient host animal for direct xenografting of fresh tumor tissue (5).

In the present study, we investigated the efficient establishment of PDX using NOG mice with clinical factors of xenotransplantation. We also discuss herein the application of this newly developed system for not only reliable preclinical studies of new anticancer drugs but also personalized anti-cancer therapies.

## Materials and methods

### Tumor tissues for transplantation

The 116 surgically removed fresh tumor tissues for transplantation were obtained at Kanagawa Cancer Center (Yokohama, Kanagawa, Japan) and Kawasaki Municipal Hospital (Kawasaki, Kanagawa, Japan) with the patients’ written informed consent for the study. The study was performed in collaboration with Keihin Coastal Area Life Innovation Comprehensive Special Zones for International Competitiveness Development (Japan) from 2011 to 2012. The ethics committees independently approved the study (authorization number: 176 at Kanagawa Cancer Center, 23–410 at Kawasaki Municipal Hospital). The entire list of engrafted tumors with the patient profiles is shown in Table I.

### Animals

NOG mice, between 6 and 12 weeks of age, were used in this study. The mice were obtained from the Central Institute for Experimental Animals (CIEA; Kanagawa, Japan) (12). All animals were housed in plastic cages (136×208×115 mm) within a vinyl isolator system (1150×500×500 mm) in a pathogen-free state, at a temperature of 22±1°C with 45±10% humidity, and a 12 h light/12 h dark cycle. All experiments involving laboratory animals were performed in accordance with the care and use guidelines of the CIEA, according to our previous studies (13–15). These guidelines meet the generally accepted international criteria on humane treatment that spare the animal needless pain and suffering, and require confirmation that the experiments conducted are of actual scientific benefit to humankind.

### Procedures for the establishment of PDX models by serial engraftment

Fresh tumor tissues were divided into three pieces under sterile conditions. One piece of each tissue specimen was immediately placed in Dulbecco’s modified minimal essential medium without antibiotics and without fetal bovine serum, and stored at 4°C until engrafting. Another piece was cryopreserved for molecular biological examination, and the last piece was fixed in 4% formaldehyde for histopathological examination. The piece for engraftment was further divided into small pieces (~8–64 mm^3^) using sterilized surgical scissors. A small incision was made in the leg of each mouse and a transplant needle was inserted until the tip reached the dorsal subcutaneous area. Approximately 10 pieces of tumor tissue were inoculated into the dorsal subcutis via the needle. After the engrafted mass expanded to over quadruple its size, the xenograft tumor was harvested and directly re-transplanted for expansion in later serial generations using the same procedure. After the tumor tissue had been passaged three times or more and histopathological examination confirmed the PDX to be a growing human tumor, we considered the PDX line as ‘established’. The established PDX tissue was divided into small pieces, completely submerged in cryopreservation medium (Cellbanker^®^1, Zenoaq, Fukushima, Japan), and then stored in liquid nitrogen. The frozen tissues were later thawed and used for experiments including re-transplantation and expansion. Mice that did not develop tumor mass over six months after engraftment were sacrificed as ‘failed’, and this was confirmed histopathologically.

### Morphological examination of the primary engrafts and the PDX descendants

For morphological analyses, sample tissues were formalin-fixed, paraffin-embedded (FFPE), sliced into 4-μm sections, and subjected to standard hematoxylin and eosin (H&E) staining or immunohistochemistry (IHC). IHC was performed using the Bond Polymer Refine Detection system (Leica Microsystems, Tokyo, Japan) according to the manufacturer’s instructions. Nuclei were counterstained with hematoxylin. Primary antibodies used for IHC were: monoclonal anti-HLA class 1-A, B, C (Hokudo, Sapporo, Japan), rabbit polyclonal anti-c-kit (Nichirei Biosciences, Tokyo, Japan); monoclonal anti-CD34, clone NU-4A1 (Nichirei Biosciences); monoclonal antileukocyte common antigen, clone PD7/26, 2B11 (CD111, Nichirei Biosciences); HER2 (Hercep Test™, Dako, Japan), monoclonal anti-estrogen receptor (ER), clone 1D5 (Nichirei Biosciences); and monoclonal antiprogesterone receptor (PgR), clone A9621A (Nichirei Biosciences). Chromogenic *in situ* hybridization (ISH) for Epstein-Barr virus (EBV)-encoded RNA (EBER) was performed using the EBER 1 DNP probe (Ventana/Roche, Tuscon, AZ, USA) and the ISH iView blue plus detection kit (Ventana/Roche) according to the provider’s instructions.

### Genetic examination of xenograft tumors in NOG mice

The exon 11 deletion mutation in the *KIT* gene in the 3rd generation xenograft of the gastrointestinal stromal tumor (GIST) was investigated as previously described (16). Briefly, DNA was extracted from the FFPE thin sections of the xenograft tumor and amplified by polymerase chain reaction (PCR) with primers: 5′-gactgagacaataattattaaaag-3′ (forward) and 5′-acccaaaaaggtgacatggaaagc-3′ (reverse). PCR products were then directly sequenced using the PCR primers and the Sanger’s method with Genetic Analyzer 3100 (Applied Biosystems/Hitachi, Japan). For EWS-FLI1 fusion mRNA detection, total RNA was extracted from the 3rd generation xenograft of the Ewing sarcoma/primitive neuroectodermal tumor (PNET), reverse transcribed to cDNA, and PCR-amplified with primers: EWS-exon 8 (5′-tcctacagccaagctccaagtc-3′) and the FLI1 exon 9 (5′-gtgatacagctggcgttggc-3′). The obtained product was directly sequenced as described for the *KIT* analysis.

### Statistical analysis

Statistical comparisons of data sets were performed by a two-sample t-test. The Chi-square test or the two-sided Fisher’s probability exact test was applied for comparisons between group frequencies. These analyses were performed using JMP version 11 software (SAS Institute Inc., Cary, NC, USA). P-values of <0.05 were considered significant.

## Results

### Efficacy of PDX line establishment in NOG mice

In total, 116 surgically removed tumor tissues were engrafted in NOG mice (Tables I and II). The group of patients who provided tumors for this study comprised 63 men and 53 women, with a mean age of 63 years. Thirty-seven tumors were obtained from primary sites and 79 tumors were from metastases. Ninety-eight tumors were epithelial (carcinomas) and 18 tumors were nonepithelial (sarcomas). Tumor specimens were engrafted on the day of surgery or 1–6 days after the surgical removal (owing to sample transport and public holidays). The primary organ site of the transplant together with the difference between the primary tumor or metastasis and the fate (established or failed) are summarized in Table III.

PDX lines were considered established when they were passaged three times or more and histopathological examination confirmed their human origin and their morphological similarity to the corresponding engrafted tumor. Of the 116 tumors engrafted, 61 were established as PDX lines, a success rate of 53%. On comparing the established cases with the failed cases, no significant differences were observed in age or gender. The average age of patients in established cases was 64 years, compared with 63 years in the failed cases and there was no statistically significant difference (P=0.53, t-test). In the established cases, 38 cases were from male patients (60%) and 23 were from female patients (43%) cases and there was also no statistically significant difference (P=0.09, Fisher’s probability exact test). High establishment rates of PDX lines were observed in tumors of the respiratory system (67%), gastrointestinal tumors (58%), and urological tumors (57%). None of the primary prostatic tumors or brain tumors yielded PDX lines in multiple trials.

The establishment rate among the primary organ sites of engrafts was different; however, there was no statistical significance (P=0.29, Chi-square test). Fifty-eight PDX lines of carcinomas (59%) and three of sarcomas (17%) were established, and the establishment rate was significantly higher for carcinomas (P<0.001, Fisher’s probability exact test). Metastatic tumors yielded PDX lines more effectively than tumors from primary sites (65% and 27%, respectively; P<0.001, Fisher’s probability exact test). Tumors engrafted into NOG mice two or more days after surgical removal showed a higher establishment rate (61%) than those engrafted on the day of surgery or the next day (51%), but there was no statistically significant difference (P=0.49, Fisher’s probability exact test).

### Preservation of the original tumor characteristics in the PDX of NOG mice

**Figure 1:**
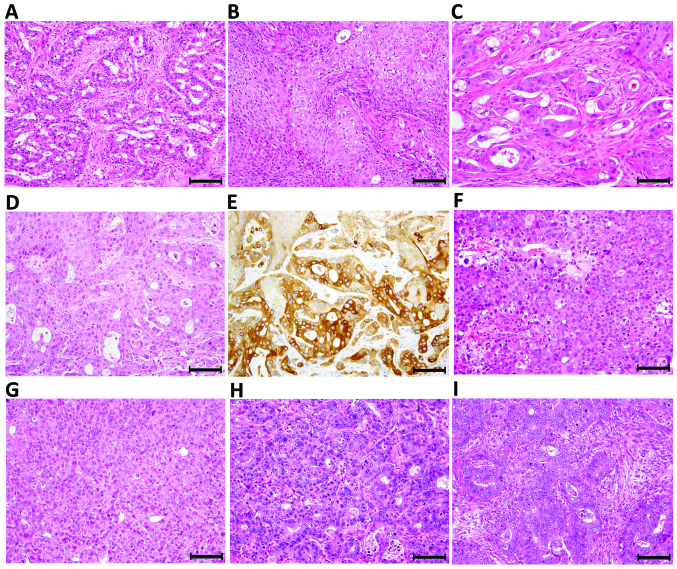
Preserved morphological characteristics observed in the xenograft tumors in NOG mice (A–E) a case of adenosquamous carcinoma of the lung, (F–G) a case of adenocarcinoma of the lung, (H–I) a case of colonic adenocarcinoma. Primary lung carcinoma contained both an adenocarcinoma component (A) and a squamous carcinoma component (B) with few transitional patterns between them. The engrafted brain metastasis showed an admixed histology of adenocarcinoma and squamous carcinoma (C) that was well preserved in the 3rd generation xenograft (D), and immunohistochemically confirmed by the detection of HLA class I (E). The engrafted tumors and the 3rd generation xenograft tumors had similar morphology in NOG PDX lines of a poorly differentiated lung adenocarcinoma (F and G) and a moderately differentiated colonic adenocarcinoma (H and I); Scale bar, 50 μm.

The morphological characteristics of the transplanted tumors, as examined by H&E-staining, were well maintained in the corresponding xenograft tumors both cellularly and structurally. One representative case of the PDX line derived from a brain metastasis of an adenosquamous carcinoma of the lung is presented in Fig. 1. The primary tumor of the lung consisted of an adenocarcinoma component and a less abundant squamous carcinoma component, and the transitional pattern between them was rare (Fig. 1A and B). In contrast, the brain metastasis tumor that was engrafted in NOG mice showed a histological structure of an admixture of adenocarcinoma and squamous carcinoma components, and the tumor characteristics were well preserved in the xenograft tumors through the 1st to the 5th generations (Fig. 1C and D). All of the components of the PDX tumor, except for the interstitium, were confirmed as having human origin by IHC for HLA class I (Fig. 1E). Similarly, a brain metastasis of a poorly differentiated adenocarcinoma of the lung was established as a PDX tumor of a poorly differentiated adenocarcinoma (Fig. 1F and G), whereas a liver metastasis of a moderately differentiated adenocarcinoma of the colon with a cribriform pattern was established as a PDX tumor with a similar histology, indicating that differentiation capacity was generally preserved (Fig. 1H and I).

**Figure 2:**
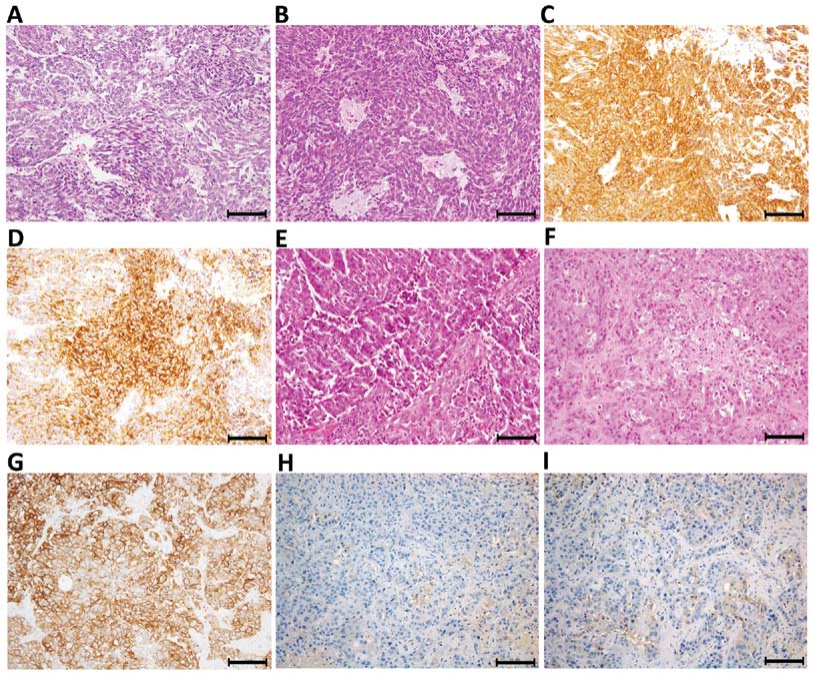
Preserved protein expression evaluated by IHC in the xenograft tumors in NOG mice (A–D) a case of GIST, (E–I) a case of breast cancer. H&E stained images of the engrafted tumor (A) and the 3rd generation xenograft tumor (B) of the established GIST case are shown. IHC for c-kit (C) and CD34 (D) gave strongly positive signals in the xenograft that reflected those in the engrafted tumor. The 3rd generation xenograft tumor of a HER2 3^+^ breast cancer case showed a similar morphology (F) and similar HER2, ER and PgR IHC patterns (G–I) to the engrafted tumor (E); Scale bar, 50 μm.

Protein expression, as examined by IHC, was also well maintained in PDX tumors. Only one PDX line was successfully established from 10 trials of GIST engraftment (Table I). The established line was derived from a recurrent metastasis after imatinib methylate treatment. The 3rd generation xenograft was examined and revealed to be strongly positive for c-kit (a proto-oncogene) and CD34 (Fig. 2A–D). A PDX line established from a brain metastasis of a HER2-positive breast cancer showed strong membranous positivity (3^+^) for HER2, and weak dispersed positivity for ER and PgR, and therefore shared the same characteristics as the engrafted tumor (Fig. 2E–I).

**Figure 3:**
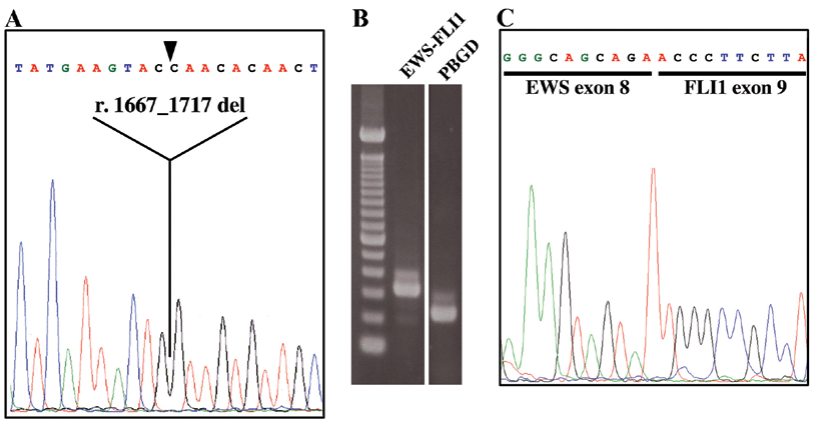
Preserved genetic alterations in the xenograft tumors in NOG mice. (A) Electropherogram of *KIT* exon 11 nucleotide sequencing for the 3rd generation xenograft tumor. A 51-bp deletion (r.1667_1717 del/p. Q556_D572 del) was detected that was identical to the alteration found in the engrafted tumor. (B) Agarose gel image of the reverse-transcription (RT)-PCR product used to amplify the EWS-FLI1 fusion mRNA. RT-PCR of the PBGD gene, a housekeeping gene, was included as a control. (C) Electropherogram of the EWS-FLI1 fusion RT-PCR product.

As expected, genetic alterations were conserved in the PDX tumors of NOG mice. The engrafted GIST tumor contained a 51-nucleotide deletion in exon 11 of *KIT* (r.1667_1717 del/p. Q556_D572 del) and DNA extracted from the 3rd generation xenograft was found to contain an identical mutation (Fig. 3A). Furthermore, the Ewing/PNET sarcoma xenograft tumor established from a brain metastasis contained the EWS-FLI1 fusion mRNA just as the original engraft (Fig. 3B and C).

### Lymphoproliferative lesion (LPL) in NOG mice

**Figure 4:**
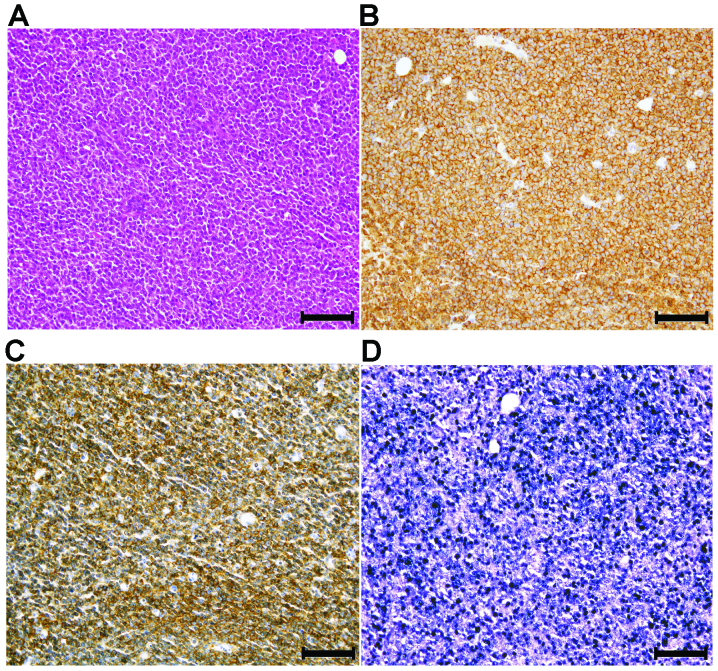
Lymphoproliferative lesion observed in the xenograft tumors. (A) A H&E-stained image of monotonous nonepithelial cells in a transplantable xenograft tumor; (B) IHC for HLA class 1; (C) IHC for CD111 (leukocyte common antigen); (D) ISH for EBER; Scale bar, 50 μm.

In eight cases (7% of all engraftments), transplantable xenograft tumors composed of large monotonous nonepithelial cells were observed whose morphology differed from that of the original tumor (Fig. 4A). This phenomenon was observed only in epithelial tumor engraftments (Tables I and II). The monotonous cells were HLA class I positive, demonstrating their human origin, and were also positive for leukocyte common antigen (CD111) by IHC and EBER by ISH, indicating the possibility that they were EBV infection-associated LPLs (Fig. 4B–D).

## Discussion

In this study, we aimed to establish a PDX line in NOG mice that preserved the original characteristics of the engrafted tumor. In a previous PDX trial in NOG mice, which included more than 300 surgically removed tumors, the establishment rate of the xenograft line was 16% (41 of 259 engrafts) for primary tumors, 31% (5 of 16 engrafts) for distant metastasis sites, and 16% (8 of 51 engrafts) for lymph node metastases (5). In this study, we achieved higher establishment rates both for primary tumors (27%) and metastatic tumors (65%). However, the constitution of the tumors used for PDX and those in the primary organ site, the ratios of primary tumors to metastatic tumors, and the numbers of each case all differed between our study and the previous study, making comparisons difficult. For example, the previous study in NOG mice used 57 primary breast cancers and obtained only three PDX lines (5%), whereas primary tumors of the breast were not included in our study (5). In fact, the establishment rate may depend on the organ site from which the engraft is taken.

Colorectal tumors showed relatively higher establishment rates than tumors from other sites in nude or SCID mice (10,11); this was also the case in the previous NOG study (17/48 engraftments, 35%) (5). In the present study, establishment rates were found to differ between sites (e.g., 58% for gastrointestinal tumors compared with 14% for urological tumors) but this difference was not statistically significant. The establishment rates of metastatic tumors were significantly higher than those of primary tumors, a finding that was consistent with previous studies (5,17,18). In our study, carcinomas showed a significantly higher establishment rate (59%) than nonepithelial tumors (17%); however, a limited number of nonepithelial tumors were tested and a larger sample size would be needed to confirm this difference.

In the present study, only subcutaneous transplantations were performed. The transplantation site has been reported to have an influence on xenograft growth (5,19–21). Considering what is known about tumor cells and microenvironmental biology, heterotopic subcutaneous tumor models seem to have some shortcomings compared with orthotopic transplantation, especially in the establishment rate and preservation of the original tumor characteristics (21). However, our subcutaneous models revealed well-preserved characteristics of the original engrafts in morphology, protein expression and gene alterations. Although further studies are needed to clarify whether this was because of the highly immunodeficient nature of NOG mice, the establishment of PDX using manageable subcutaneous transplantations is convenient when compared with skillful orthotopic transplantation.

Unexpectedly, in the present study, we experienced no significant difference in the establishment rate of PDX lines between the tumors engrafted early (on the day of surgery or the next day) and the tumors engrafted after 2 days. To the best of our knowledge, no previous studies have investigated this issue. One could speculate that the so-called cancer stem cells responsible for tumorigenicity in mice might be resistance to the severe stress induced by removal from the patients. Although further investigation is needed, this information might help oncology researchers to improve and simplify PDX line establishment, particularly in light of our findings that subcutaneous transplantation is not inferior to orthotopic transplantation with regard to preserving the original engraft characteristics.

The high occurrence of LPL was the most problematic aspect of the establishment of NOG mice-PDX, which arose because of the severely immunodeficient nature of the animal model. In eight cases (7% of all engraftments), the engrafted tumors were replaced by LPL until the 3rd generation of xenografts. The LPLs were demonstrated to be EBV infection-associated, as has been previously reported (20,22,23). Fujii and colleagues also reported that EBV-infected B cells originating from the donor were distributed systemically within the NOG mouse (23). LPLs were transplantable, and difficult to distinguish from the proper xenografts in terms of gross appearance. It is therefore important that histology of xenografts is checked before transplantation into new mice. However, the frequency of LPLs is acceptable when considering the merits of NOG mice. Replacement of the engrafted tumors by LPL accounted for 15% of the failed cases in PDX line establishment, indicating that the major cause of failed cases is therefore likely to be the nature of the xenografts.

Owing to progression in the field of oncology, the demand for relevant human tumor models is increasing. *In vivo* models play a vital role in the extrapolation of data to human patients, especially in the development of anticancer agents. Evidence that both tumor differentiation and tumor structure were highly conserved between the original surgical specimen and the PDX tumor confirms the suitability of our mouse model for the study of tumor biology. Applications of this model, not only for more common tumors, but also for uncommon tumors, such as sarcomas or pediatric tumors, will provide researchers with reliable comparative preclinical data that may contribute to the development of novel cancer therapies. The rapid and efficient establishment of PDX linking with clinical information may lead in the future to the development of personalized anticancer therapies by simulating various treatments in individual PDX mice, so-called cancer xenopatients.

## Figures and Tables

**Table I:** The entire list of patients from which the engrafted tumors were taken and the fate of the xenografts.

No.	Age	Gender	Original tumor site	Pathology	Primary/Metastasis	Tumor type	Result
1	43	M	Lung	Adenosquamous carcinoma	Brain metastasis	Epithelial	Established
2	60	M	Lung	Adenocarcinoma	Brain metastasis	Epithelial	Established
3	69	M	Lung	Adenocarcinoma	Brain metastasis	Epithelial	Failed^c^
4	35	F	Large intestine	Tubular adenocarcinoma	Liver metastasis	Epithelial	Established
5	51	F	Breast	Ductal carcinoma	Brain metastasis	Epithelial	Established
6	62	M	Prostate	Adenocarcinoma	Primary	Epithelial	Failed
7	65	M	Large intestine	Tubular adenocarcinoma	Liver metastasis	Epithelial	Established
8	76	F	Large intestine	Tubular adenocarcinoma	Liver metastasis	Epithelial	Established
9	60	F	Lung	Adenocarcinoma	Brain metastasis	Epithelial	Failed^c^
10	66	F	Lung	Adenocarcinoma	Brain metastasis	Epithelial	Established
11	74	F	Large intestine	Tubular adenocarcinoma	Liver metastasis	Epithelial	Established
12	58	M	Nerve	MPNST	Primary	Mesenchymal	Established
13	28	M	Bone	Ewing/PNET	Brain metastasis	Mesenchymal	Established
14	58	M	Thyroid	Papillary carcinoma	Brain metastasis	Epithelial	Failed
15	76	M	Large intestine	Tubular adenocarcinoma	Liver metastasis	Epithelial	Failed^c^
16	65	F	Breast	Ductal carcinoma	Brain metastasis	Epithelial	Failed
17	69	F	Large intestine	Tubular adenocarcinoma	Brain metastasis	Epithelial	Established
18	71	F	Large intestine	Tubular adenocarcinoma	Liver metastasis	Epithelial	Established
19	74	M	Esophagus	Squamous cell carcinoma	Brain metastasis	Epithelial	Established
20	68	M	Kidney	Renal cell carcinoma	Brain metastasis	Epithelial	Established
21	81	F	Lung	Adenocarcinoma	Brain metastasis	Epithelial	Failed^c^
22	80	M	Small intestine	GIST	Primary	Mesenchymal	Failed
23	55	M	Prostate	Adenocarcinoma	Primary	Epithelial	Failed
24	65	F	Breast	Ductal carcinoma	Brain metastasis	Epithelial	Failed
25	51	M	Large intestine	Tubular adenocarcinoma	Liver metastasis	Epithelial	Established
26	66	M	Pancreas	Ductal carcinoma	Primary	Epithelial	Failed
27	40	F	Brain	Glioblastoma	Primary	Mesenchymal	Failed
28	61	M	Large intestine	Tubular adenocarcinoma	Liver metastasis	Epithelial	Established
29	43	M	Brain	Glioblastoma	Primary	Mesenchymal	Failed
30	60	M	Stomach	GIST	Peritoneal metastasis	Mesenchymal	Established
31	77	M	Stomach	Tubular adenocarcinoma	Peritoneal metastasis	Epithelial	Established
32	46	M	Brain	Astrocytoma	Primary	Mesenchymal	Failed
33	61	F	Duodenum	Tubular adenocarcinoma	Primary	Epithelial	Failed
34	65	F	Breast	Ductal carcinoma	Brain metastasis	Epithelial	Failed
35	64	M	Large intestine	Tubular adenocarcinoma	Liver metastasis	Epithelial	Established
36	69	F	Lung	Squamous cell carcinoma	Brain metastasis	Epithelial	Established
37	69	F	Uterus body	Adenocarcinoma	Brain metastasis	Epithelial	Established
38^a^	58	F	Large intestine	Tubular adenocarcinoma	Liver metastasis	Epithelial	Established
39	70	F	Large intestine	Tubular adenocarcinoma	Brain metastasis	Epithelial	Established
40	47	M	Primary unknown	Adenocarcinoma	Brain metastasis	Epithelial	Established
41	71	F	Large intestine	Tubular adenocarcinoma	Liver metastasis	Epithelial	Established
42	71	F	Uterus body	Adenocarcinoma	Brain metastasis	Epithelial	Failed^c^
43	55	F	Large intestine	Tubular adenocarcinoma	Liver metastasis	Epithelial	Established
44	52	M	Lung	Small cell carcinoma	Brain metastasis	Epithelial	Established
45	68	M	Prostate	Adenocarcinoma	Primary	Epithelial	Failed
46	73	M	Lung	Adenocarcinoma	Brain metastasis	Epithelial	Failed
47	73	M	Large intestine	Tubular adenocarcinoma	Liver metastasis	Epithelial	Established
48	55	F	Breast	Ductal carcinoma	Brain metastasis	Epithelial	Failed
49	68	M	Stomach	Tubular adenocarcinoma	Brain metastasis	Epithelial	Failed^c^
50	52	M	Large intestine	GIST	Primary	Mesenchymal	Failed
51	53	F	Nerve	MPNST	Primary	Mesenchymal	Failed
52	64	M	Large intestine	Tubular adenocarcinoma	Brain metastasis	Epithelial	Failed
53	62	F	Pancreas	Ductal carcinoma	Primary	Epithelial	Established
54^b^	70	M	Pancreas	Ductal carcinoma	Lymph node metastasis	Epithelial	Failed
55^b^	70	M	Pancreas	Ductal carcinoma	Lymph node metastasis	Epithelial	Established
56^b^	70	M	Pancreas	Ductal carcinoma	Lymph node metastasis	Epithelial	Established
57	35	F	Stomach	GIST	Primary	Mesenchymal	Failed
58	64	M	Lung	Large cell carcinoma	Brain metastasis	Epithelial	Established
59	74	F	Pancreas	Anaplastic carcinoma	Primary	Epithelial	Failed
60	71	F	Pancreas	Ductal carcinoma	Primary	Epithelial	Established
61	74	F	Pancreas	Ductal carcinoma	Primary	Epithelial	Failed
62	70	M	Kidney	Transitional cell carcinoma	Brain metastasis	Epithelial	Failed
63	53	F	Large intestine	Tubular adenocarcinoma	Liver metastasis	Epithelial	Failed
64	85	F	Stomach	GIST	Primary	Mesenchymal	Failed
65	67	M	Kidney	Renal cell carcinoma	Brain metastasis	Epithelial	Established
66	82	M	Lung	Adenocarcinoma	Brain metastasis	Epithelial	Established
67	61	M	Kidney	Renal cell carcinoma	Peritoneal metastasis	Epithelial	Established
68	70	F	Stomach	GIST	Primary	Mesenchymal	Failed
69	64	F	Brain	Glioblastoma	Primary	Mesenchymal	Failed
70	49	M	Pancreas	Ductal carcinoma	Primary	Epithelial	Failed
71	61	F	Lung	Adenocarcinoma	Brain metastasis	Epithelial	Established
72	70	F	Large intestine	Tubular adenocarcinoma	Liver metastasis	Epithelial	Failed^c^
73	72	F	Large intestine	GIST	Primary	Mesenchymal	Failed
74	69	F	Stomach	GIST	Primary	Mesenchymal	Failed
75	67	F	Large intestine	Tubular adenocarcinoma	Liver metastasis	Epithelial	Established
76	79	M	Brain	Glioblastoma	Primary	Mesenchymal	Failed
77	60	M	Large intestine	Tubular adenocarcinoma	Liver metastasis	Epithelial	Established
78	63	F	Gallbladder	Pleomorphic carcinoma	Brain metastasis	Epithelial	Failed^c^
79	37	F	Large intestine	Tubular adenocarcinoma	Liver metastasis	Epithelial	Failed
80	70	M	Large intestine	Tubular adenocarcinoma	Liver metastasis	Epithelial	Established
81	68	F	Stomach	GIST	Primary	Mesenchymal	Failed
82	63	M	Large intestine	Tubular adenocarcinoma	Liver metastasis	Epithelial	Failed
83	70	F	Large intestine	Tubular adenocarcinoma	Liver metastasis	Epithelial	Failed
84	60	F	Large intestine	Tubular adenocarcinoma	Liver metastasis	Epithelial	Failed
85	56	M	Large intestine	Tubular adenocarcinoma	Liver metastasis	Epithelial	Established
86	58	F	Breast	Ductal carcinoma	Brain metastasis	Epithelial	Established
87	16	F	Stomach	GIST	Primary	Mesenchymal	Failed
88	62	M	Large intestine	Tubular adenocarcinoma	Liver metastasis	Epithelial	Failed
89	71	M	Pancreas	Ductal carcinoma	Primary	Epithelial	Established
90	65	F	Kidney	Renal cell carcinoma	Skin metastasis	Epithelial	Failed
91	51	M	Pancreas	Ductal carcinoma	Primary	Epithelial	Failed
92	51	F	Large intestine	Tubular adenocarcinoma	Liver metastasis	Epithelial	Failed
93	75	M	Lung	Adenocarcinoma	Brain metastasis	Epithelial	Failed
94	80	M	Pancreas	Ductal carcinoma	Primary	Epithelial	Failed
95	77	M	Kidney	Renal cell carcinoma	Skin metastasis	Epithelial	Established
96	68	M	Large intestine	Tubular adenocarcinoma	Liver metastasis	Epithelial	Established
97	63	M	Pancreas	Ductal carcinoma	Primary	Epithelial	Failed
98	61	F	Pancreas	Ductal carcinoma	Primary	Epithelial	Failed
99	67	F	Pancreas	Ductal carcinoma	Primary	Epithelial	Established
100	61	M	Lung	Adenocarcinoma	Brain metastasis	Epithelial	Established
101	71	M	Stomach	Tubular adenocarcinoma	Brain metastasis	Epithelial	Established
102^a^	59	F	Large intestine	Tubular adenocarcinoma	Liver metastasis	Epithelial	Established
103	63	M	Large intestine	Tubular adenocarcinoma	Liver metastasis	Epithelial	Established
104	61	F	Thyroid	Follicular carcinoma	Brain metastasis	Epithelial	Established
105	71	M	Pancreas	Ductal carcinoma	Primary	Epithelial	Established
106	46	M	Large intestine	Mucinous adenocarcinoma	Liver metastasis	Epithelial	Established
107	66	M	Pancreas	Ductal carcinoma	Primary	Epithelial	Established
108	80	F	Large intestine	Tubular adenocarcinoma	Liver metastasis	Epithelial	Established
109	65	M	Pancreas	Ductal carcinoma	Primary	Epithelial	Established
110	81	F	Thyroid	Anaplastic carcinoma	Primary	Epithelial	Established
111	71	M	Pancreas	Ductal carcinoma	Primary	Epithelial	Established
112	54	F	Breast	Ductal carcinoma	Brain metastasis	Epithelial	Failed
113	62	M	Large intestine	Tubular adenocarcinoma	Liver metastasis	Epithelial	Established
114	68	M	Lung	Adenocarcinoma	Brain metastasis	Epithelial	Established
115	63	M	Large intestine	Tubular adenocarcinoma	Brain metastasis	Epithelial	Established
116	78	M	Kidney	Renal cell carcinoma	Brain metastasis	Epithelial	Failed

a Different operations of the same patient,

b independent metastatic lesions of the same operation,

c replaced by lymphoproliferative lesions, M, male; F, female; MPNST, malignant peripheral nerve sheath tumor; GIST, gastrointestinal stromal tumor.

**Table II:** Summary of the engrafted tumors and the fate of xenografts.

Engrafted tumor information		Established		Failed (LPL)		Total	
			
Organ site	Type	Primary	Metastasis	Primary	Metastasis	Primary	Metastasis
Gastrointestinal							
Esophagus	Epithelial	0	1	0	0	0	1
Stomach	Epithelial	0	2	0	1 (1)	0	3
	Mesenchymal	0	1	6	0	6	1
Small intestine	Epithelial	0	0	1	0	1	0
	Mesenchymal	0	0	1	0	1	0
Large intestine	Epithelial	0	25	0	10 (2)	0	35
	Mesenchymal	0	0	2	0	2	0
Other digestive							
Pancreas	Epithelial	8	2	8	1	16	3
Gallbladder	Epithelial	0	0	0	1 (1)	0	1
Respiratory							
Lung	Epithelial	0	10	0	5 (3)	0	15
Breast and female genital							
Breast	Epithelial	0	2	0	5	0	7
Uterus	Epithelial	0	1	0	1 (1)	0	2
Urologic							
Kidney	Epithelial	0	4	0	3	0	7
Neurologic							
Brain	Mesenchymal	0	0	5	0	5	0
Nerve	Mesenchymal	1	0	1	0	2	0
Others							
Bone	Mesenchymal	0	1	0	0	0	1
Thyroid	Epithelial	1	1	0	1	1	2
Prostate	Epithelial	0	0	3	0	3	0
Primary unknown	Epithelial	0	1	0	0	0	1

LPL, lymphoproliferative lesions.

**Table III:** Comparison of the establishment rate of xenograft lines.

	Established	Failed	Total	%	P-value
Original tumor sites					0.29^a^
Gastrointestinal	29	21	50	58	
Other digestive	10	10	20	50	
Respiratory	10	5	15	67	
Breast and female	3	6	9	33	
genital					
Urological	4	3	7	57	
Neurological	1	6	7	14	
Others	4	4	8	50	
Total	61	55	116	53	
Tumor type					<0.001^b^
Carcinomas	58	40	98	59	
Sarcomas	3	15	18	17	
Tumor site					<0.001^b^
Primary	10	27	37	27	
Metastasis	51	28	79	65	
Time to engraftment^c^					0.49^b^
Early	47	46	93	51	
Delayed	14	9	23	61	

a Chi-square test,

b two-sided Fisher’s probability exact test,

c engraftment on the day of surgery or the next day was considered ‘early’, and engraftment after 2 days was considered as ‘delayed’.
